# An emerging role for cyclic dinucleotide phosphodiesterase and nanoRNase activities in *Mycoplasma bovis*: Securing survival in cell culture

**DOI:** 10.1371/journal.ppat.1008661

**Published:** 2020-06-29

**Authors:** Xifang Zhu, Eric Baranowski, Yaqi Dong, Xixi Li, Zhiyu Hao, Gang Zhao, Hui Zhang, Doukun Lu, Muhammad A. Rasheed, Yingyu Chen, Changmin Hu, Huanchun Chen, Eveline Sagné, Christine Citti, Aizhen Guo

**Affiliations:** 1 The State Key Laboratory of Agricultural Microbiology, College of Veterinary Medicine, Huazhong Agricultural University, Wuhan, China; 2 Hubei International Scientific and Technological Cooperation Base of Veterinary Epidemiology, International Research Center for Animal Disease of Ministry of Science and Technology of China, Wuhan, China; 3 Key Laboratory of Preventive Veterinary Medicine in Hubei Province, The Cooperative Innovation Center for Sustainable Pig Production, Wuhan, China; 4 Key Laboratory of Development of Veterinary Diagnostic Products, Key Laboratory of Ruminant Bio-products, Ministry of Agriculture and Rural Affairs of China, Wuhan, China; 5 IHAP, Université de Toulouse, INRAE, ENVT, Toulouse, France; Miami University, UNITED STATES

## Abstract

Mycoplasmas are host-restricted prokaryotes with a nearly minimal genome. To overcome their metabolic limitations, these wall-less bacteria establish intimate interactions with epithelial cells at mucosal surfaces. The alarming rate of antimicrobial resistance among pathogenic species is of particular concern in the medical and veterinary fields. Taking advantage of the reduced mycoplasma genome, random transposon mutagenesis was combined with high-throughput screening in order to identify key determinants of mycoplasma survival in the host-cell environment and potential targets for drug development. With the use of the ruminant pathogen *Mycoplasma bovis* as a model, three phosphodiesterases of the DHH superfamily were identified as essential for the proliferation of this species under cell culture conditions, while dispensable for axenic growth. Despite a similar domain architecture, recombinant Mbov_0327 and Mbov_0328 products displayed different substrate specificities. While rMbovP328 protein exhibited activity towards cyclic dinucleotides and nanoRNAs, rMbovP327 protein was only able to degrade nanoRNAs. The Mbov_0276 product was identified as a member of the membrane-associated GdpP family of phosphodiesterases that was found to participate in cyclic dinucleotide and nanoRNA degradation, an activity which might therefore be redundant in the genome-reduced *M*. *bovis*. Remarkably, all these enzymes were able to convert their substrates into mononucleotides, and medium supplementation with nucleoside monophosphates or nucleosides fully restored the capacity of a Mbov_0328/0327 knock-out mutant to grow under cell culture conditions. Since mycoplasmas are unable to synthesize DNA/RNA precursors *de novo*, cyclic dinucleotide and nanoRNA degradation are likely contributing to the survival of *M*. *bovis* by securing the recycling of purines and pyrimidines. These results point toward proteins of the DHH superfamily as promising targets for the development of new antimicrobials against multidrug-resistant pathogenic mycoplasma species.

## Author summary

Mycoplasmas are among the simplest self-replicating organisms. Pathogenic species are of particular concern in the medical and veterinary fields given the alarming rate of antimicrobial resistance documented in these simple, but fast-evolving bacteria. With the use of the ruminant pathogen *Mycoplasma bovis* as a model, several proteins participating in the degradation of cyclic dinucleotides and short RNA molecules were found critical for the survival of this pathogen when grown in the presence of host cells. Remarkably, these essential functions can become dispensable upon the addition of nucleotides into the host-cell culture medium. Since mycoplasmas are unable to synthesize DNA/RNA precursors *de novo*, cyclic dinucleotide and nanoRNA degradation are likely contributing to the survival of *M*. *bovis* by securing the recycling of purines and pyrimidines. While illustrating the pivotal role played by nutrients in the mycoplasma host-cell interplay, this study unveils strategies used by reduced-genome bacteria to overcome their limited metabolic capacities and secure their survival in highly sophisticated hosts. These results identified promising targets for the development of new antimicrobials against multidrug-resistant pathogenic mycoplasma species.

## Introduction

Mycoplasmas are among the simplest prokaryotes capable of self-replication [1]. These organisms belong to the class *Mollicutes*, a large group of wall-less bacteria whose evolution from Gram-positive ancestors resulted in a severe genome reduction. Over these last years, mycoplasmas have gained particular attention since their minimal genome provided a valuable platform to address fundamental biological questions [2–6]. Pathogenic species, in turn, can be viewed as simplified models for the study of host-microbe interactions.

Despite their apparent simplicity, mycoplasmas are successful pathogens capable of establishing persistent infections and causing debilitating diseases in humans and a wide range of animal hosts [1, 7]. Many pathogenic species are causing growing concerns in both the medical and veterinary fields due to intrinsic antibiotic resistance and a rapid decrease in susceptibility to chemotherapeutic agents [8–11]. Genomic studies have considerably improved our understanding of the structure and dynamics of these organisms [7], but provided only limited information on the molecular determinants shaping their pathogenic properties. The main reason is the lack of a classical repertoire of virulence genes in pathogenic species. Thus, mycoplasmas are likely to have evolved specific mechanisms to access nutrients from the host that do not rely on sophisticated machineries, such as secretion systems found in more complex bacteria. A notable exception is a group of proteins encoded by mobile elements in mycoplasmas that share similarities with type IV secretion systems [12–15]. These elements are responsible for massive exchanges of chromosomal material within and across mycoplasma species and contribute to the horizontal dissemination of new phenotypic traits [13, 15–17]. The paucity of secretion systems in mycoplasmas is consistent with the limited number of toxins so far identified in pathogenic species. The only example is the CARDS toxin produced by the human pathogen *Mycoplasma pneumoniae* [18]. Hydrogen peroxide and hydrogen sulfide are currently the only cytotoxic metabolic compounds found to be produced by these organisms [19]. This situation contrasts with the sophisticated mechanisms that mycoplasmas have evolved to escape the immune responses [20]. They include efficient genetic systems of antigenic variation and sophisticated machinery found to capture and cleave immunoglobulins [20, 21].

Because of their limited metabolic capacities, mycoplasmas are fastidious organisms to grow in axenic conditions. However, the development of specific culture media for several pathogenic species has dramatically facilitated their propagation in the laboratory and the selection of high-passage strains with attenuated virulence [22, 23]. Unfortunately, the accumulation of genomic alterations during this long-term evolutionary process only provided limited information on the molecular basis of the avirulent phenotype. Nevertheless, mutations in cytoadherence genes are repeatedly found in avirulent strains indicating that mycoplasma adhesion to host cells is crucial for infectivity [24–26]. The paucity of genetic tools to manipulate mycoplasmas is another limitation to the study of virulence factors in pathogenic species. Over the last few years, synthetic biology has emerged as a powerful technology in mycoplasmology [4, 27, 28]. This technology has been successfully applied to several phylogenetically related species but is facing some limitations with other species, including the ruminant pathogens *Mycoplasma agalactiae* and *Mycoplasma bovis* [29]. For these species, random transposon mutagenesis, when combined with relevant screening systems, remains an efficient strategy for functional genomics studies [30–33]

*M*. *bovis* is a re-emerging cause of pneumonia and mastitis in cattle worldwide, whose management is challenging because of the ineffectiveness of current vaccines and the high level of resistance to antibiotic treatments. In the present study, we used this pathogenic species as a model system to identify key factors regulating the proliferation of mycoplasmas under co-cultivation with host cells. Growth-deficient mutants were isolated by combining transposon mutagenesis with high-throughput screening in cell culture. The genetic and phenotypic characterization of selected mutants identified members of the DHH superfamily involved in cyclic dinucleotide and nanoRNA degradation as critical for *M*. *bovis* proliferation under co-cultivation with embryonic bovine lung (EBL) cells.

## Results

### Characterization of the *M*. *bovis* mutant library

A library of 2285 individual mutants was generated by random transposon mutagenesis, and mutants displaying a growth-deficient phenotype upon co-incubation with EBL cells were selected. The library was constructed by transformation with the non-replicative plasmid pMT85 [34], which encodes a modified Tn4001 (mTn). Since mTn contains no transposase gene, its chromosomal insertion is stable in addition to conferring gentamicin resistance [30]. DNA sequence analysis of 1032 mutants randomly selected from the library revealed a broad distribution of unique mTn insertions in the HB0801 chromosome (S1 Fig). Coding sequences (CDSs) found disrupted in selected mutants reached about 41% of *M*. *bovis* coding capacity (857 CDS in HB0801). This value is in agreement with experimental estimations of the minimal size of a library to reach saturation mutagenesis of all nonlethal insertion sites [6, 35].

### Functional analysis of the *M*. *bovis* reduced genome under cell culture conditions

**Fig 1:**
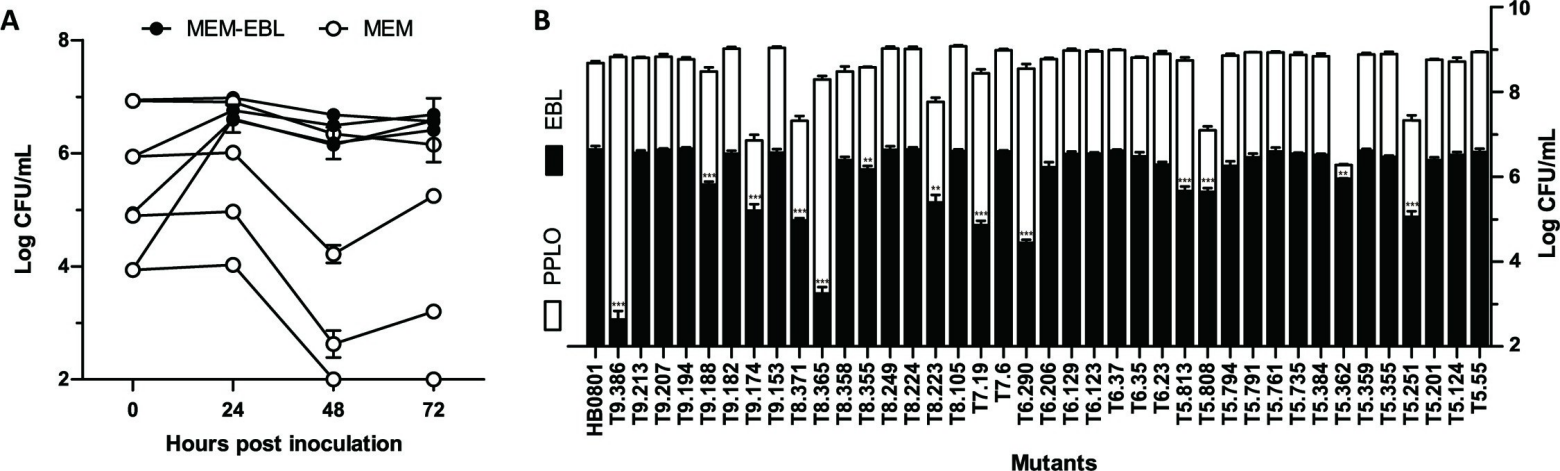
Growth-deficient phenotype of *M*. *bovis* mutants under cell culture conditions. (A) Growth curves of strain HB0801 under co-cultivation with host cells. Serial dilutions of culture stocks were inoculated to EBL cells seeded at a density of 2 x 10^4^ cells/cm^2^ (MEM-EBL) or incubated in the cell culture medium alone (MEM). (B) Growth phenotype of *M*. *bovis* and selected mutants under axenic (PPLO) and cell culture (EBL) conditions. Mycoplasma titers were determined after 72 h of incubation. The data are presented as the means of three independent assays. Standard deviations are indicated by error bars. *p* values are indicated by asterisks (**p < 0.01; ***p < 0.001). Differences were considered to be significant at *p* < 0.01.

The one megabase genome of *M*. *bovis* strain HB0801 was used to map the genomic regions that are critical for mycoplasma survival in cell culture. Growth-deficient mutants were selected upon co-incubation with EBL cells using a high-throughput screening strategy developed for *M*. *agalactiae*, a close relative of *M*. *bovis* [30]. Growth curve experiments were conducted with HB0801 to rule out any influence of the multiplicity of infection on mycoplasma titers reached at the end of the co-incubation with EBL cells (Fig 1A). The moderate increase in mycoplasma titers observed at 72h in MEM medium alone is likely to be stimulated by the release of nutrients from the inoculum, such as DNA, which was recently identified as a limiting factor for *M*. *bovis* proliferation under cell culture conditions [32]. By using this screening strategy, 39 mutants were identified and further characterized by comparing their proliferation under axenic (PPLO medium) and cell culture conditions (Fig 1B). Six mutants were found unable to reach wild-type mycoplasma titers upon co-cultivation with EBL cells while producing titers similar to the parental strain in axenic conditions (Fig 1B and Table 1). DNA sequencing revealed single mTn insertions in the chromosome of these mutants that mapped within six CDS related to DNA replication, recombination, and repair (COG1466, Mbov_0528; COG3611, Mbov_0751; and COG0507, Mbov_0848), signal transduction mechanisms (COG3887, Mbov_0276), and nucleotide transport and metabolism (COG0618, Mbov_0328), as well as proteins with unknown functions (Mbov_0804) (Table 1).

**Table 1:** Mapping of transposon insertions in selected *M*. *bovis* mutants.

Mutant ^a^	Genomic position (orientation) ^b^	CDS ^c^	CDS position (orientation) ^d^	CDS identity based on conserved domains ^e^
T5.813	887280 (+)	Mbov_0751	0.04 (-)	Replication initiation and membrane attachment protein DnaB
T6.290	318377 (+)	Mbov_0276	0.77 (+)	DHH superfamily, c-di-AMP phosphodiesterase GdpP
T7.19	624675 (+)	Mbov_0528	0.69 (+)	DNA polymerase III subunit delta (HolA)
T8.365	989394 (+)	Mbov_0848	0.94 (-)	Exodeoxyribonuclease V alpha subunit (RecD)
T9.188	939150 (-)	Mbov_0804	0.39 (-)	DUF2779 domain-containing protein
T9.386	388367 (+)	Mbov_0328	0.46 (-)	DHH superfamily, bifunctional nanoRNase/pAp phosphatase NrnA

^a^ Mutants were designated according to transformation and clone numbers.

^b^ Transposon insertion sites were defined based on the published sequence (GenBank accession number CP002058). The orientation of the mTn is indicated in parenthesis.

^c^ CDSs found disrupted in *M*. *bovi*s mutants are indicated by the mnemonic codification in the GenBank database.

^d^ For each CDS, the relative position and orientation of the mTn are indicated.

^e^ Conserved domains were identified using the Basic Local Alignment Search Tool (https://blast.ncbi.nlm.nih.gov/Blast.cgi). DUF: Domain of unknown function.

### The Mbov_0328/0327 locus is critical for *M*. *bovis* growth in cell culture

**Fig 2:**
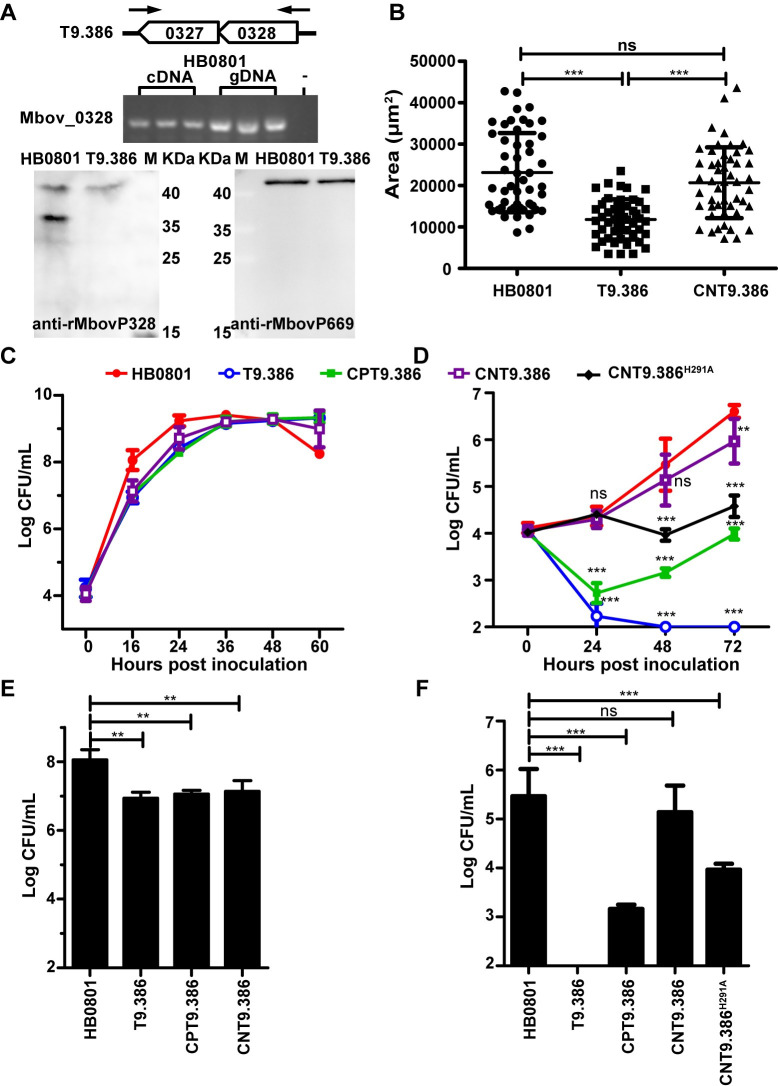
The Mbov_0328/0327 locus is essential for *M*. *bovis* proliferation in cell culture. (A) Representation of the Mbov_0328/0327 locus, including oligonucleotide primers, used for PCR amplification of HB0801 cDNA and gDNA (upper panel) and Western blot analysis of Mbov_0328 expression in mutant T9.386 (lower panel). (B) Comparative analysis of mycoplasma colony size. Cultures of parental (HB0801), mutant (T9.386), and complemented (CNT9.386) strains were spotted onto PPLO agar plates and grown for 5 days at a 37°C under 5% CO_2_. Colony sizes (μm^2^) were determined under a light microscope using CellScan software (32× magnification). Given the heterogeneity of mycoplasma colonies, mean values were determined by analyzing 50 individual colonies per strain. Standard deviations are indicated by error bars. ***p < 0.001; ns = *p* > 0.05. (C-F) Growth phenotype of the parental strain (HB0801), mutant T9.386 (T9.386), and mutant T9.386 transformed with plasmid pCP-T9.386 (CPT9.386) or pCN-T9.386 (CNT9.386), as well as pCN-T9.386^H291A^ (CNT9.386 ^H291A^). Growth under axenic conditions (C) and mycoplasma titers reached at 16 h in axenic PPLO medium (E). Growth under cell culture conditions (D) and mycoplasma titers reached at 48 h in cell culture (F). The data are presented as the means of three independent assays. Standard deviations are indicated by error bars. *p* values are indicated by asterisks (**p < 0.01, ***p < 0.001, ns = *p* > 0.05).

The extreme phenotype exhibited by T9.386 prompted us to characterize this mutant further. The disruption of Mbov_0328 in T9.386 was confirmed by Western blotting using a specific antiserum (see Materials and Methods) (Fig 2A). This result was consistent with proteomic data (see below) indicating no peptide corresponding to Mbov_0328 products in the mutant, except for the region located upstream of the mTn insertion site. Interestingly, peptides corresponding to Mbov_0327 products were significantly down-regulated in T9.386 suggesting that the mTn inserted in Mbov_0328 is also influencing downstream gene expression. RT-PCR amplification revealed that these contiguous CDSs are co-transcribed as a single mRNA (Fig 2A). Complementation studies with T9.386 demonstrated the role of Mbov_0328/0327 in *M*. *bovis* growth under cell culture conditions. The mutant was transformed with plasmids pCP-T9.386 and pCN-T9.386, two derivatives of plasmid pOH/P (see Materials and Methods) having the Mbov_0328/0327 coding region either (i) inserted under the control of a constitutive promoter (pCP-T9.386) or (ii) surrounded by upstream and downstream non-coding regions (NCRs) of the locus (pCN-T9.386). Growth curve experiments confirmed the growth-deficient phenotype of T9.386 upon co-cultivation with EBL cells (Fig 2D and 2F). They further revealed a limited delay at the early stage of T9.386 proliferation in axenic PPLO medium (Fig 2C and 2E), as well as a smaller colony size upon development onto solid medium (Fig 2B). While transformation with pCP-T9.386 and pCN-T9.386 had no apparent influence on T9.386 growth in PPLO medium (Fig 2C and 2E), both plasmids were able to dramatically improve the proliferation of T9.386 in cell culture (Fig 2D and 2F) and development onto solid medium (Fig 2B). However, only pCN-T9.386 was able to restore wild-type growth. As expected, transformation with the control plasmid pOH/P did not influence T9.386 or HB0801 proliferation in PPLO and cell culture (S2 Fig). These data confirmed that functions essential for *M*. *bovis* growth under cell culture conditions are encoded by the Mbov_0328/0327 locus and revealed the occurrence of regulatory regions in surrounding NCRs.

### Mbov_0328/0327 mediates cyclic dinucleotides and nanoRNAs degradation in *M*. *bovis*

**Fig 3:**
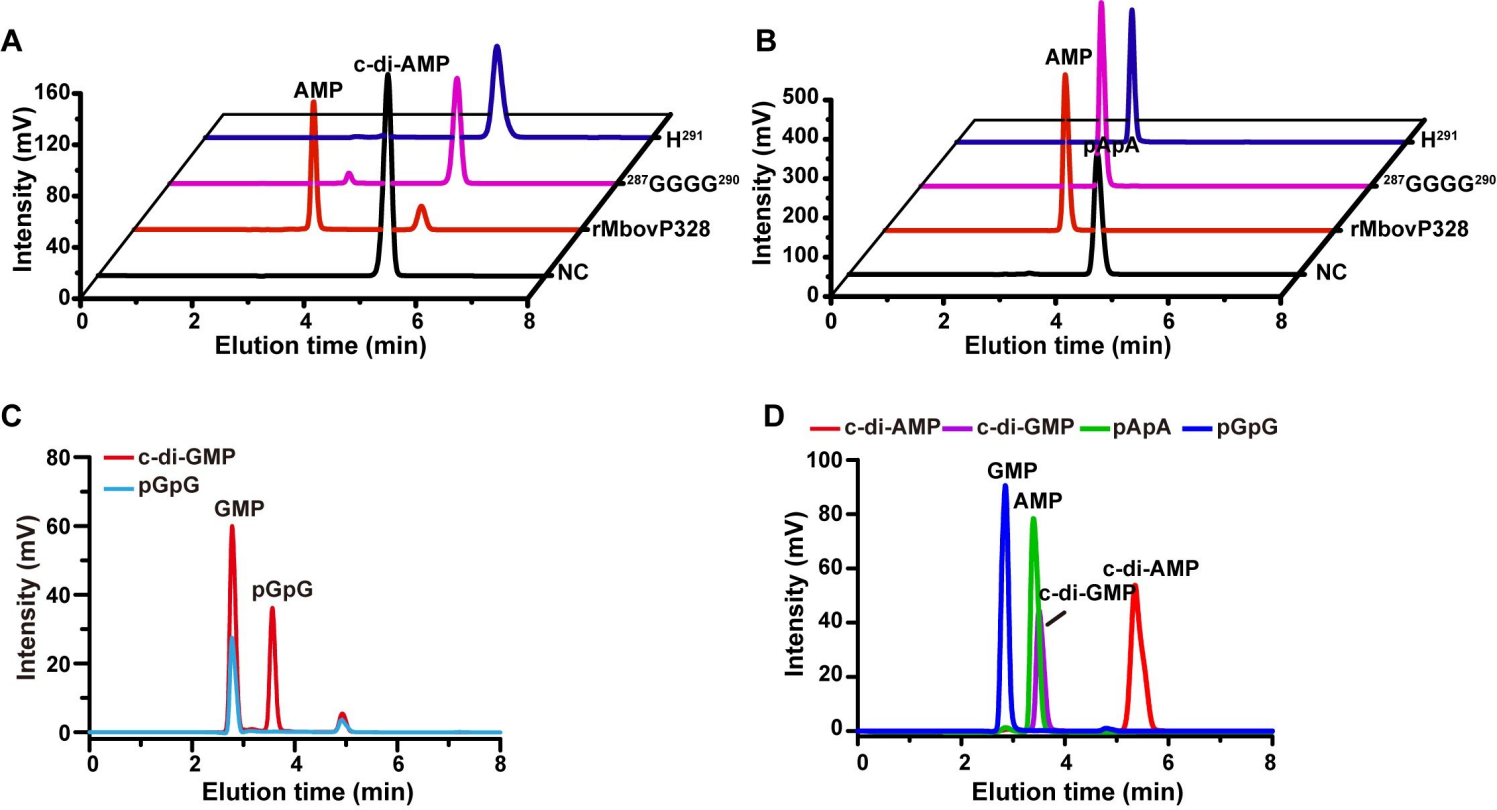
Biochemical characterization of the *M*. *bovis* phosphodiesterases Mbov_0327 and Mbov_0328. (A) Analysis of c-di-AMP hydrolysis by rMbovP328 and mutated version of rMbovP328. (B) Analysis of pApA hydrolysis by rMbovP328 and mutated version of rMbovP328. NC, negative control. The assay conditions are described in Materials and Methods. (C) Analysis of c-di-GMP and pGpG hydrolysis by rMbovP328. (D) HPLC analysis of the hydrolysis of c-di-AMP, c-di-GMP, pApA, and pGpG by rMbovP327.

Proteins encoded by Mbov_0327 and Mbov_0328 share 61% global similarity and possess the typical features of the NrnA bifunctional nanoRNase/pAp phosphatase of the DHH superfamily. Multiple sequence alignments with representative members of this superfamily revealed a similar architecture with the presence of a conserved DHH motif and a type 1 DHH associated (DHHA1) domain (S3 Fig). The DHH/DHHA1 subfamily encompasses a large group of phosphodiesterases with broad substrate specificities. Typical substrates of DHH/DHHA1 phosphodiesterases include single-stranded DNA, nanoRNA (RNA oligonucleotides ≤ 5 nucleotides), and cyclic dinucleotides [36–38]. To assess the enzymatic activity associated with Mbov_0327 and Mbov_0328, we produced His-tagged recombinant proteins and tested their activities against naturally occurring cyclic dinucleotides and nanoRNAs. Upon incubation with rMbovP328 (recombinant Mbov_0328 product), HPLC analysis demonstrated the conversion of c-di-AMP into AMP (Fig 3A). This activity was optimal at pH 6.5–7.5 in the range of 37–50°C, with a preference for Mn^2+^ and Co^2+^ and an optimal Mn^2+^ concentration of 5 mM (S4 Fig). Interestingly, rMbovP328 was also found to be active on c-di-GMP, leading to the accumulation of pGpG and GMP (Fig 3C). These data suggest that rMbovP328 can hydrolyze both c-di-AMP and pApA to AMP and, to a lesser extent, c-di-GMP and pGpG to GMP. This promiscuous substrate specificity is a typical feature of most characterized DhhP-type phosphodiesterases [38]. The term “DhhP” was originally used to designate a DHH-DHHA1 domain protein in *Borrelia burgdorferi* [39]. The degradation activity of rMbovP328 against pApA and pGpG confirmed its nanoRNase activity (Fig 3B and 3C). Site-directed mutagenesis was used to confirm the role of conserved residues GGGGH (287–291) in the activity of rMbovP328. Two mutated versions of the protein were constructed in which amino acid residues 287 to 290 (GGGG) and 291 (H) were changed to alanine. These amino acid substitutions were all found to abrogate the activity of rMbovP328 in degrading c-di-AMP but not pApA (Fig 3A and 3B). Unlike rMbovP328, the enzymatic characterization of rMbovP327 (recombinant Mbov_0327 product) revealed only a nanoRNase activity against pApA and pGpG, but not cyclic dinucleotide degradation activity (Fig 3D). Altogether, these data indicate that Mbov_0327 and Mbov_0328 products possess different activities, despite similar domain architectures. While rMbovP328 exhibited activity towards cyclic dinucleotides and nanoRNAs, rMbovP327 was only able to degrade nanoRNAs.

### Cyclic dinucleotide phosphodiesterase and nanoRNase activities are redundant in *M*. *bovis*

**Fig 4:**
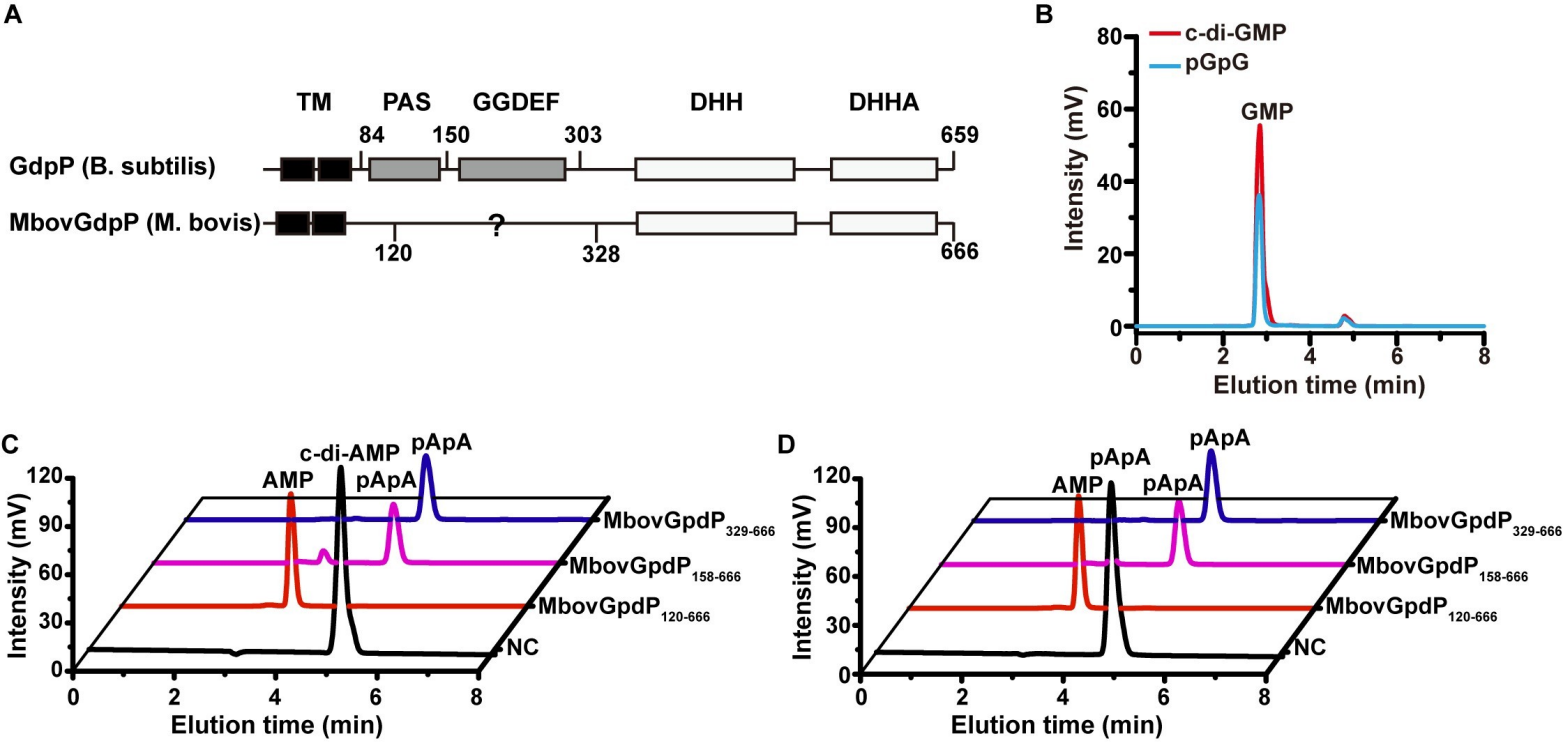
Biochemical characterization of the *M*. *bovis* DHH domain protein MbovGdpP. (A) Analysis of conserved domain between typical GdpP and rMbovGdpP. (B) Analysis of c-di-GMP and pGpG hydrolysis by rMbovGdpP_120-666_. (C and D) Enzymatic activity of truncated rMbovGdpP against c-di-AMP (C) and pApA (D). NC, negative control.

The pivotal role played by DHH phosphodiesterases in the interaction of *M*. *bovis* with host cells was further supported by the disruption of Mbov_0276 in one of the six selected mutants (see mutant T6.290 in Table 1). Indeed, Mbov_0276 encodes a putative c-di-AMP phosphodiesterase of the GdpP superfamily.https://www.ncbi.nlm.nih.gov/Structure/cdd/cddsrv.cgi?uid=cl34688 Typical GdpP are membrane-associated DHH/DHHA1-type phosphodiesterases that carry a sensory PAS domain and a degenerated GGDEF domain (Fig 4A) [36–38]. To study the activity associated with Mbov_0276, we produced a soluble recombinant version of the protein, further designated as rMbovGdpP_120-666_, by deleting the predicted N-terminal transmembrane region (amino acids 1 to 119). Enzymatic assays confirmed the phosphodiesterase activity of rMbovGdpP_120-666_ and revealed broad substrate specificity. Indeed, the protein was able to degrade c-di-AMP and c-di-GMP, as well as pApA and pGpG, to AMP and GMP, respectively (Fig 4B to 4D). Several truncated versions of rMbovGdpP were produced to map functional domains. They included rMbovGdpP_158-666_ and rMbovGdpP_329-666_ in which the deletion of the N-terminal region was extended to amino acid 157 and 328, respectively. HPLC analysis revealed that truncated proteins were still able to convert c-di-AMP to pApA but failed to produce AMP suggesting no nanoRNase activity (Fig 4C). This was further confirmed by incubating truncated proteins with pApA that generated no detectable products (Fig 4D). These data indicate that the DHH/DHHA1 domains in rMbovGdpP are essential for the cyclic dinucleotide phosphodiesterase activity, while nanoRNase activity also required amino acid region 120–157. Attempts to complement the growth-deficiency of mutant T6.290 were unsuccessful, and further studies are needed to fully understand the biological importance of the different DHH phosphodiesterases encoded by this genome-reduced mycoplasma species.

### Nucleotide recycling is essential for *M*. *bovis* growth in cell culture

**Fig 5:**
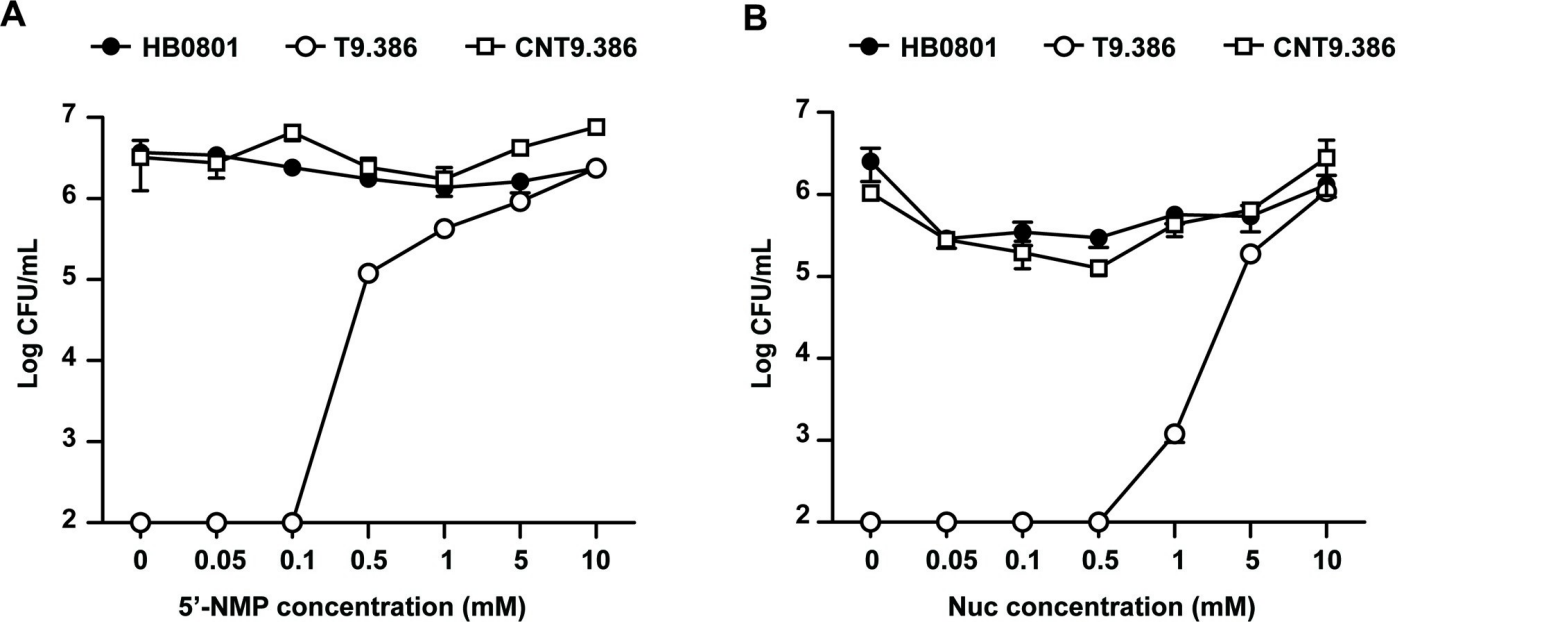
The Mbov_0328/0327 locus is required for nucleotide production in *M*. *bovis*. (A and B) Nucleotide production is important for *M*. *bovis* proliferation under cell co-culture conditions. *M*. *bovis* parental strain (HB0801), mutant T9.386 (T9.386), and mutant T9.386 transformed with plasmid pCN-T9.386 (CNT9.386) were co-cultivated with EBL cells. The cell culture medium was supplemented with increasing concentration of NMPs (A) or nucleosides (B), and mycoplasma titers were determined at 72 h. Mycoplasma titers were determined at each 24 h interval. The data are presented as the means of three independent assays. Standard deviations are indicated by error bars.

Despite differences in substrate specificities, DHH phosphodiesterases characterized in this study all contribute to nucleotide recycling in *M*. *bovis*. This prompted us to investigate the influence of nucleotides on T9.386 growth in cell culture. Remarkably, medium supplementation with NMPs (AMP, GMP, CMP, and UMP) or nucleosides (adenosine, guanosine, cytidine, and uridine) dramatically enhanced the growth of T9.386 (Fig 5). The mutant nearly reached wild-type mycoplasma titers at NMP and nucleoside concentrations of 1 and 5 mM, respectively. As expected, supplementation with NMPs or nucleosides had no or only limited influence on mycoplasma titers reached by the parental strain HB0801 and the complemented strain CNT9.386 (Fig 5). These results indicate that NMP production associated with the Mbov_0328/0327 locus is essential for *M*. *bovis* survival under cell culture conditions. To further investigate the role of Mbov_0327 and Mbov_0328 in nucleotide recycling, we transformed mutant T9.386 with a derivative of pCN-T9.386 in which the amino acid change H291A was introduced in Mbov_0328 to inactivate its cyclic dinucleotide phosphodiesterase activity. The resulting construct pCN-T9.386^H291A^ is thus only able to restore the nanoRNase activity encoded by Mbov_0328 and Mbov_0327. RT-PCR amplification confirmed the transcription of the mutated Mbov_0328 in transformant CNT9.386^H291A^ (S5 Fig). Growth curve experiments revealed an intermediate phenotype for CNT9.386^H291A^, with mycoplasma titers at 72h post-inoculation ca. 100-fold lower than reached by CNT9.386 (Fig 2D). These data suggest that NMP production contributed by degradation of both cyclic dinucleotides and nanoRNAs is critical for *M*. *bovis* survival under cell culture conditions.

### Mbov_0328/0327 disruption in *M*. *bovis* induces global changes in protein expression

**Fig 6:**
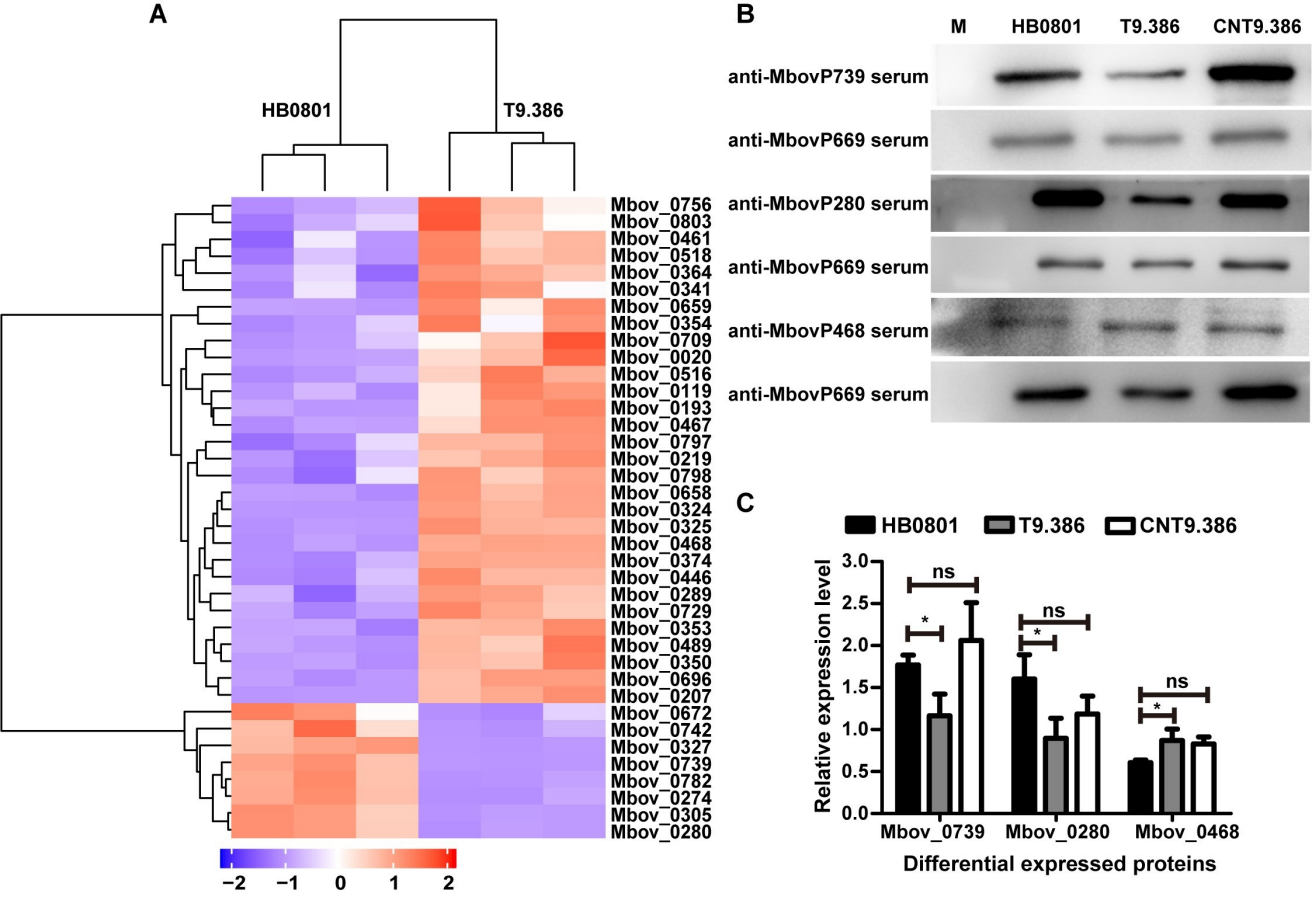
Differential expression profiles of *M*. *bovis* HB0801 and mutant T9.386 based on whole-cell proteomics. (A) A heatmap showing the 38 proteins with a significant difference in expression (difference ≥ 2-folds; *p* < 0.05) between the parental (HB0801) and the T9.386 (T9.386) strains. Each row represents one protein designated by corresponding CDS in *M*. *bovis* HB0801. Each column represents one sample (three replicates per strain). The dendrogram at the top of the figure clusters samples based on similarity in protein expression patterns. The dendrogram at the left of the figure shows relationships in proteins based on expression profiling. Red color represents a higher relative protein abundance compared with the mean, while blue illustrates a relative abundance below the mean. (B) Western blot analysis of Mbov_0280, Mbov_0468 and Mbov_0739 expression in *M*. *bovis* parental strain (HB0801), mutant T9.386 (T9.386) and mutant T9.386 transformed with plasmid pCN-T9.386 (CNT9.386). Mbov_0669 was used as control. Anti-sera raised against individual *M*. *bovis* recombinant proteins were used as primary antibodies (1:500). (C) The relative expression level of Mbov_0280, Mbov_0468, and Mbov_0739. Values were normalized using Mbov_0669. The data are presented as the means of three independent assays. Standard deviations are indicated by error bars. *p* values are indicated by asterisks (*p < 0.05, ns = *p* > 0.05).

Since influence of Mbov_0328/0327 disruption originates from the lack of NMP products, to further understand the influence of Mbov_0328/0327 disruption in *M*. *bovis*, we used LC-MS/MS to characterize changes in the protein expression profiles of T9.386 and HB0801 in PPLO axenic medium with abundant Nucs or NMPs, in which both strains are able to grow similarly. A total of 600 proteins were detected, representing ca. 80% of the total number of CDS in HB0801(S6 Fig). Differential expression analysis identified 38 proteins in T9.386, with 30 up-regulated and 8 down-regulated (Fig 6A and S1 Table). The differential expression of one up-(MbovP468) and two down-regulated (MbovP280 and MbovP739) proteins was confirmed by Western blotting, which further documented their wild-type expression in the complemented strain CNT9.386 (Fig 6B). Relative expression levels were calculated using Mbov_0669 product as a reference (Fig 6C), since proteomic data failed to reveal any difference in its expression. The main category of proteins differentially expressed in T9.386 are of unknown function with no similarity outside of the *Mollicutes*, of which ca. 60% were putative membrane-associated proteins (S1 Table). According to functional domains and KEGG pathways analyses, a broad range of biological processes were found impacted in T9.386, including transport, metabolism, and structural maintenance of chromosomes (S1 Table). Interestingly, two putative transmembrane proteins up-regulated in T9.386 share similarity with multidrug efflux transporters of the MATE-like (Mbov_0193) and MFS-3 (Mbov_0756) superfamilies. These multidrug transporters were recently associated with bacterial resistance to antimicrobials [40–43], suggesting that Mbov_0328/0327 might also influence *M*. *bovis* susceptibility to antibiotics, in addition to securing survival in cell culture. Altogether these data indicate that Mbov_0328/0327 disruption may induce important biological modifications in *M*. *bovis*. Whether increased levels of c-di-AMP are responsible for these modifications remains to be further investigated.

## Discussion

The development of specific culture media has dramatically facilitated the study of fastidious mycoplasmas. These media were developed to overcome the limited metabolic capacity of these genome-reduced organisms and meet their important nutritional requirements. These laboratory conditions have largely contributed to the understanding of the biology of these minimal bacteria but provided only limited information on the strategies these organisms have evolved to secure their survival in the animal host and get access to essential nutritional resources. In the absence of small animal models, eukaryotic cell cultures as re-emerged as a valuable environment to revisit the minimal cell concept at the light of host-pathogen interactions [30–33, 44]. Our functional genomic study with the genome-reduced pathogen *M*. *bovis* revealed that phosphodiesterases involved in cyclic dinucleotides and nanoRNAs degradation are critical for survival under cell culture conditions, while dispensable in axenic medium. Remarkably, the essential nature of these enzymatic activities can be easily bypassed by *M*. *bovis* when nucleotides are added to the culture medium. These results suggest that nucleotide recycling in genome-reduced bacteria is critical for survival in the host-cell environment, and shed new light on the biological activity of DHH phosphodiesterases in bacteria.

### DHH/DHHA1-type phosphodiesterases in the genome-reduced *M*. *bovis*

The DHH superfamily is characterized by conserved DHH and DHH associated (DHHA) domains. Members of this superfamily include cyclic dinucleotide phosphodiesterase of the DhhP and GdpP types, as well as NrnA bifunctional nanoRNase/pAp phosphatase and RecJ exonuclease [36–38]. DhhP-type phosphodiesterases are present in almost all c-di-AMP synthesizing bacteria, including the human pathogen *Mycoplasma pneumoniae* [45]. Most characterized DhhP-type phosphodiesterases hydrolyze both c-di-AMP and pApA to AMP, as well as c-di-GMP and pGpG to GMP [38]. The broad substrate specificity of DhhP-type enzymes overlaps the activity of NrnA, which can degrade nanoRNAs (2- to 5-mer oligoribonucleotides) [36]. NrnA can be found mainly in bacterial species that do not have Orn oligoribonuclease orthologs. Among these bacteria is a large group of bacteria that includes the mycoplasmas as well as many of the Firmicutes [45, 46]. *M*. *pneumoniae* NrnA (MPN140) is closely related to Mbov_0327 and Mbov_0328 products, with a global similarity of 61% and 57%, respectively. However, only the Mbov_0327 product displayed typical features of nanoRNase with no activity on cyclic dinucleotides. In contrast, the Mbov_0328 product was identified as a cyclic dinucleotide DhhP-type phosphodiesterase and a functional homolog of *M*. *pneumoniae* PdeM (MPN549). Cyclic di-AMP is an essential second messenger in many bacteria that was recently identified in *M*. *pneumoniae* [36, 45, 47]. In this species, c-di-AMP is produced by the diadenylate cyclase CdaM (MPN244) and degraded by the phosphodiesterase PdeM (MPN549) [45]. These proteins are conserved in *M*. *bovis*, with Mbov_0496 product sharing 58% global similarity with CdaM and Mbov_0328 product being the functional homolog of PdeM. Cyclic di-AMP is not only essential for bacteria that produce it but also toxic upon accumulation [36]. Functional genomic studies with *M*. *pneumoniae* confirmed the essentiality of CdaM and PdeM [5, 45]. While essential in cell culture, Mbov_0328 was found dispensable under axenic conditions. The molecular mechanisms responsible for the conditional essentiality of Mbov_0328 remains to be elucidated, but the occurrence of multiple DHH phosphodiesterase genes in *M*. *bovis* might secure its c-di-AMP hydrolyzing activity. This hypothesis is supported by the lack of a GdpP-type phosphodiesterase in *M*. *pneumoniae*, a unique feature shared by all the members of the pneumoniae phylogenetic group that encompasses several human pathogenic species.

### The emerging role of nucleotides recycling in genome-reduced mycoplasmas

The growth-promoting effect of nucleotides and nucleosides on Mbov_0328/0327 knock-out mutants suggests that nucleotide recycling is essential for *M*. *bovis* survival under cell culture conditions. Thus, cyclic dinucleotides and nanoRNAs degradation activities identified in the present study likely contribute to nucleotide recycling in genome-reduced mycoplasmas that are well known for having limited metabolic capacities. This emerging role of nucleotides recycling is supported by several functional genomic studies with *M*. *bovis* and other ruminant mycoplasma species that repeatedly documented the disruption of the purine salvage adenine phosphoribosyltransferase as detrimental for mycoplasma growth in cell culture [30–32, 44]. The recent identification of extracellular DNA as a limiting nutrient for *M*. *bovis* growth and cytotoxicity in cell culture also raises questions regarding the contribution of nucleotides recycling to virulence [32]. Many pathogenic bacteria possess a membrane-associated GdpP-type phosphodiesterase whose activity is required for virulence [48–50]. These enzymes carry a heme-binding PAS domain that can positively or negatively influence their phosphodiesterase activity [36]. The heme-binding activity of MbovGdpP (Mbov_0276 product) remains to be demonstrated, but the close proximity of Mbov_0276 to a putative hemolysin gene (Mbov_0279) is a good indication of the possible role of MbovGdpP in sensing heme signals during *M*. *bovis* infection. The DhhP-type phosphodiesterase encoded by Mbov_0328 provides *M*. *bovis* with a second protein to regulate c-di-AMP levels. Cyclic dinucleotide phosphodiesterases are indeed essential in bacteria that rely on only a single enzyme for c-di-AMP degradation, such as *B*. *burgdorferi* and *M*. *pneumoniae* [39, 45]. In other bacteria, increased c-di-AMP levels can be associated with important phenotypic alterations, such as severe growth defects, abnormal cell morphology, poor osmotic stress tolerance, and reduced virulence [36, 38]. In *M*. *bovis*, the different DHH family members identified in the present study likely contribute to c-di-AMP homeostasis, with small colony size being the only phenotype exhibited by the Mbov_0328 knock-out mutant in axenic conditions. Whether increased c-di-AMP levels in *M*. *bovis* may contribute to growth defects in cell culture is not known, but nucleotide recycling was found essential for survival in this environment. Since mycoplasmas are unable to synthesize DNA/RNA precursors *de novo*, cyclic dinucleotide phosphodiesterase and nanoRNase activities are thus pivotal in the recycling of purines and pyrimidines.

While illustrating the central role played by nutrients in the mycoplasma host-cell interplay, this study unveils strategies used by reduced-genome bacteria to overcome their limited metabolic capacities and secure their survival in highly sophisticated hosts. These results identified members of the DHH superfamily as promising targets for the development of new antimicrobials against multidrug-resistant pathogenic mycoplasma species.

## Materials and methods

### Ethics statement

Animal experiments were approved by the Ethics Committee of Huazhong Agricultural University (approval no. SYXK(ER) 2015–0084) and conducted in accordance with the Hubei Regulations for the Administration of Affairs Concerning Experimental Animals issued in 2005.

### Bacterial strains and culture conditions

*M*. *bovis* strain HB0801 (reference number M2010040; China Center for Type Culture Collection, Wuhan University, Wuhan, China) was cultured at 37°C in PPLO broth as previously described [51]. When needed, gentamicin (100 μg/mL) or puromycin (10 μg/mL) was added to the medium. Mycoplasma cultures were stored at -80°C, and colony-forming units (CFUs) were determined as previously described [30]. *E*. *coli* strains DH5α and BL21 (DE3) (TransGen Biotech, Beijing, China) were cultured at 37°C in Luria–Bertani (LB) broth and used for DNA cloning and protein expression, respectively. EBL cells, kindly provided by Prof. Fei Xue, were grown in minimum essential medium (MEM) supplemented with 10% heat-inactivated fetal calf serum (Gibco, Grand Island, NY, USA) at 37°C under an atmosphere of 5% CO_2_/95% air.

### DNA constructions and oligonucleotide primers

DNA constructions and oligonucleotide primers used in the present study are listed in S2 Table. Plasmid pOH/P was used as a backbone for complementation studies [16]. This plasmid was derived from p20-1miniO/T by replacing (i) the *tetM* region by the *pac* gene encoding a puromycin N‐acetyltransferase [12], and (ii) the replication origin of *M*. *agalactiae* by its counterpart in HB0801 [33, 51]. The plasmid pCP-T9.386 contains a pOH/P backbone in which the Mbov_0328/0327 coding region was inserted under the control of the *M*. *agalactiae* P40 lipoprotein promoter [30]. The DNA construction pCN-T9.386 is similar to pCP-T9.386 but contains no promoter, and the Mbov_0328/0327 coding region was extended to surrounding NCRs. Finally, pCN-T9.386^H291A^ is a derivative of pCN-T9.386 in which Mbov_0328 codon 291 was modified to introduce a substitution from histidine to alanine (H291A). The modified sequence was synthesized at Beijing Tianyi Huiyuan Bioscience & Technology, Inc. DNA constructions used for *M*. *bovis* protein expression in *Escherichia coli* (see below) were synthesized at Beijing Tianyi Huiyuan Bioscience & Technology, Inc. UGA tryptophan codons in *M*. *bovis* were changed into UGG in order to avoid premature stop codons in *E*. *coli*. DNA was amplified by PCR using the Phusion High-Fidelity DNA Polymerase (New England Biolabs, MA, USA).

### Transposon mutagenesis in *M*. *bovis*

Mycoplasma cells were transformed with plasmid pMT85 using polyethylene glycol (PEG). *M*. *bovis* cultures at the late-log phase were washed in Dulbecco’s phosphate-buffered saline (Invitrogen Corporation, Carlsbad, CA, USA) and resuspended in 0.1 M CaCl_2_ at a concentration of 10^9^ CFU/mL. Competent cells (10^8^ CFU) were mixed with 3 μg of pMT85 and 10 μg of yeast tRNA and transferred in 1 mL of 50% PEG8000 (Sigma–Aldrich Corporation, St. Louis, MO, USA). After incubation for 1 min, the mixture was diluted in 5 mL of non-selective PPLO medium and incubated at 37°C for 3 hours. Then, mycoplasma cells were washed and resuspended in 1 mL of PPLO medium and plated onto a selective PPLO solid medium. After incubation at 37°C for 3 to 7 days, single colonies were picked and grown in 1 mL of selective PPLO broth.

### Co-cultivation of *M*. *bovis* with EBL cells

*M*. *bovis* and EBL cells were co-cultivated in MEM supplemented with 2 mM L-glutamine and Earle’s balanced salts. EBL cells were seeded at a density of 2 × 10^4^ cells/cm^2^ in 24-well plates and inoculated with *M*. *bovis* at various multiplicities of infection. Mycoplasma and EBL cells were allowed to grow at 37°C under an atmosphere of 5% CO_2_/95% air. At different times post-inoculation, mycoplasma titers were determined by CFU titrations following one freeze-thaw (-80°C/+37°C) cycle to release intracellular bacteria. *M*. *bovis* growth-deficient mutants were selected using a high-throughput method [30]. Briefly, EBL cells were seeded in the wells of 96-well plates at a density of 2 × 10^4^ cells/cm^2^. Individual mutants were inoculated to EBL cells using a 96-pin replicator (Nunc, Thermo Fisher Scientific, Rockford, IL, USA). *M*. *bovis* mutants and EBL cells were co-cultivated for 72 h at 37°C under 5% CO_2_ in the presence of gentamicin (100 μg/mL). Growth-deficient mutants were identified by spotting freeze-thawed co-cultures onto solid culture medium. Culture stocks of *M*. *bovis* mutants were used as controls. The development of mycoplasma colonies was observed under a light microscope after 3 to 5 days of incubation at 37°C. *M*. *bovis* mutants that failed to produce detectable CFUs upon co-cultivation with EBL cells, but produced detectable CFUs in PPLO, were selected.

### Identification of transposon insertion sites in *M*. *bovis* chromosomal DNA

Genomic DNA was extracted from mycoplasma cells using the Genomic DNA Extraction kit (TaKaRa Biotechnology (Dalian) Co., Ltd., Dalian, China). For each mutant, the position of the mTn insertion in the *M*. *bovis* chromosome was determined by sequencing the junction between the *M*. *bovis* genomic DNA and the 5’- or 3’-end of the transposon using linear amplification-mediated PCR as described previously [52]. The *M*. *bovis* genome was fragmented by digestion with *Nsi*I. The primers listed in S2 Table were used to amplify the targeted fragment. The amplified PCR products were sequenced at Beijing Tianyi Huiyuan Bioscience & Technology, Inc. (Beijing, China).

### Bioinformatics analysis

The genome sequence of *M*. *bovis* reference strains HB0801 (GenBank accession no. CP002058) was retrieved from the GenBank database. Transposon insertion sites were mapped on the chromosome of *M*. *bovis* using the basic local alignment search tool for nucleotides (BLASTn; https://blast.ncbi.nlm.nih.gov/Blast.cgi?PAGE_TYPE=BlastSearch) and Geneious prime software (https://www.geneious.com/academic/). Predicted functional domains were identified with BLAST protein (https://blast.ncbi.nlm.nih.gov/Blast.cgi?PAGE=Proteins). Protein alignments were performed using ClustalW multiple sequence alignment programs (https://www.ebi.ac.uk/Tools/msa/clustalw2/) combined with ESPript 3.0 software (http://espript.ibcp.fr/ESPript/ESPript/). Metabolic pathways were analyzed using the Kyoto Encyclopedia of Genes and Genomes (KEGG) database.

### Production and purification of *M*. *bovis* recombinant proteins

Recombinant proteins used in the present study are listed in S2 Table. DNA constructions were cloned into pET28b (+) and transferred into *E*. *coli* BL21 cells for protein expression as previously described [33]. Briefly, protein expression was induced with 0.8 mM isopropyl-β*-*D-thiogalactose (IPTG) for 20 h at 16°C or 4 h at 37°C. Cultures were then collected and resuspended in binding buffer (2 mM imidazole, 20 mM Na_3_PO_4_, 500 mM NaCl; pH 7.4) and homogenesis at 1000 bar (4°C) for 3–4 times. Soluble proteins were purified by nickel affinity chromatography (GE Healthcare, Piscataway, NJ, USA) after centrifuge at 12,000 x g for 30 min. Purified proteins were analyzed by SDS-PAGE, and protein concentrations were determined by BCA protein assay (Thermo Fisher Scientific, Waltham, MA, USA).

### Enzymatic activity assays

The phosphodiesterase and nanoRNase activities of *M*. *bovis* recombinant proteins were assayed by using c-di-AMP and c-di-GMP as cyclic dinucleotide substrates, and pApA and pGpG as oligoribonucleotide substrates. Chemicals were purchased from BIOLOG Life Science Institute (Bremen, Germany). The assays were conducted at 37°C in 10 mM Tris-HCl (pH 7.0) and 2.5 mM MnCl_2_ using 10 μM recombinant protein and 50 μM c-di-NMP or 500 μM oligoribonucleotides. The reaction was stopped by boiling for 10 min and insoluble materials were removed by centrifugation at 20000 x *g*. Soluble materials were analyzed by reversed-phase high-pressure liquid chromatography (HPLC) (Shimadzu Corporation, Kyoto, Japan) using a RP-C18 column (4.6 × 250 mm, 5 μm; Thermo Fisher Scientific, Waltham, MA, USA). Samples were eluted using 90% phosphate buffer (pH 6.0) containing 30 mM K_2_HPO_4_, 20 mM KH_2_PO_4_, and 10% methanol [53]. The reaction products were monitored by measuring the absorbance at 254 nm. Temperature, metal, and pH dependence was determined with the use of c-di-AMP as substrate.

### Production of mouse antisera, and Western blot analysis

Four-week-old BALB/c mice were used to generate polyclonal antibodies against *M*. *bovis* recombinant protein rMbovP328. Five animals were immunized with recombinant proteins (100 μg) emulsified in an equal volume of Freund's complete adjuvant (Sigma–Aldrich Corporation) for the first immunization and Freund's incomplete adjuvant (Sigma–Aldrich Corporation) for the others. Immunization was performed by subcutaneous injections at 2-week intervals. Sera raised against recombinant proteins rMbovP280, rMbovP468, rMbovP669, rMbovP739 were produced by using the same procedure using soluble recombinant proteins purified from *E*. *coli* (S2 Table).

Western blot analysis was carried out with the total cell protein content (50μg) derived from *M*. *bovis* cultures grown in PPLO for 36 h [54]. Proteins were separated by SDS-PAGE and transferred onto a nitrocellulose membrane (EMD Millipore Corporation, Billerica, MA, USA). Mouse antiserum (1:500) raised against *M*. *bovis* recombinant proteins was used as a primary antibody and a horseradish peroxidase-conjugated goat anti-mouse IgG antibody (1:5000) was used as the secondary antibody. Western blots were developed with the use of an enhanced chemiluminescence substrate kit (Thermo Fisher Scientific).

### Profiles of differentially expressed proteins

The expression profiles of differentially expressed proteins of *M*. *bovis* were determined by liquid chromatography-tandem mass spectrometry (LC-MS/MS). Mycoplasma cells were harvested from 200 mL of PPLO culture by centrifugation at 8000 x *g* for 20 min. Cells were washed extensively with phosphate-buffered saline before resuspension in 200 μL of ice-cold lysis buffer (4% SDS, 1 mM dithiothreitol, 100 mM Tris-HCl pH 7.6). The solution was homogenized using a Fastprep-24 automated homogenizer (MP Biomedicals LLC, Santa Ana, CA, USA) and lysed by sonication and boiling for 15 min. Whole-cell proteins were quantified by using the BCA Protein Assay Kit (Bio-Rad, Hercules, CA, USA) and samples (20 μL) were separated by SDS-PAGE and digested to peptides using the filter-aided sample preparation method as described previously [55]. Peptides were separated using an RP-C18 column (10 × 75 μm, 3 μm; Thermo Fisher Scientific) and eluted with buffer A (0.1% formic acid) and buffer B (84% acetonitrile, 0.1% formic acid) at a flow rate of 300 nL/min. LC-MS/MS analysis was performed using an Easy-nLC system (Thermo Fisher Scientific) coupled to a Q Exactive Hybrid Quadrupole-Orbitrap Mass Spectrometer (Thermo Fisher Scientific). MS was performed in positive ion mode. Survey scans were acquired at a resolution of 70,000 with a m/z ratio of 200 and a ranging scan range of 300–1800 m/z. The resolution for MS was set to 17,500 at m/z = 200. The generated data were identified and quantified by using the MaxQuant quantitative proteomics software package (Max Planck Institute of Biochemistry, Am Klopferspitz, Germany) as described previously [56]. Each protein group was set up with 3 biological replicates and the average LFQ intensities were compared as ratios for the comparison of altered expressed proteins. Differences were considered to be significant when *p* < 0.05 while difference ≥ 2 folds. The mass spectrometry proteomics data have been deposited to the ProteomeXchange Consortium via the PRIDE [57] partner repository with the dataset identifier PXD017374.

### Statistical analyses

Statistical analysis were performed using Student's *t*-test for one comparison and one-way ANOVA for multiple comparison for data in normal distribution, while Mann–Whitney U test for one comparison with SPSS software (SPSS, Inc., Chicago, IL, USA). Student's *t*-test and Mann–Whitney U test was performed in selected mutants, one-way ANOVA was used in cell colonies, *M*. *bovis* growth and protein expression level, the significance among groups was evaluated by using Post Hoc test (Tukey’s test). Significance values obtained were presented as *p* * < 0.05, *p* ** < 0.01, *p* *** < 0.001 and “ns” represented non-significance (*p* > 0.05).

The numerical data used in all figures are included in S1 Data.

## Supporting information

S1 Fig Mapping of transposon insertions in the *M*. *bovis* chromosome. Schematic representation illustrating the distribution of unique mTn insertions in the HB0801 chromosome. Data are derived from sequence analysis of 1032 mutants. Transposons inserted into the coding sequences and non-coding regions are indicated by black and red lines, respectively. Coding sequences on the HB0801 chromosome are represented by blue arrows. Pink and orange colors indicate tRNA and rRNA regions, respectively.
(PPTX)

S2 Fig Growth phenotype of parental and mutant strain transformed with the control plasmid pOH/P (CHB0801 and CT9.386). *M*. *bovis* CHB0801 and CT9.386 were generated following transformation of parental strain HB0801 and mutant T9.386 with the control plasmid pOH/P. (A) Growth phenotype of CHB0801 and CT9.386 in PPLO medium. Mycoplasmas (10^4^ CFUs) were grown in 1 ml of PPLO medium. (B) Growth phenotype of CHB0801 and CT9.386 in cell culture. Mycoplasmas (10^4^ CFUs/ml) were inoculated to EBL cells seeded at a density of 2 x 10^4^ cells/cm^2^. Mycoplasma titers were determined at different time post-inoculation. The data are presented as the means of three independent assays. Standard deviations are indicated by error bars.
(PPTX)

S3 Fig Multiple sequence alignment of Mbov327 and Mbov328. The alignments of *M*. *bovis* Mbov327 and Mbov328, *Mycobacterium tuberculosis* Rv2837c (PDB code 5CET), *M*. *pneumoniae* MPN140 (UniProtKB entry P75144) and MPN549 (UniProtKB entry P75229), *Borrelia burgdorferi* DhhP (UniProtKB entry O51564), and *Mycobacterium smegmatis* MSMEG_2630 (PDB code 4LS9) were performed using ESPript 3.0. The secondary structure of Mbov328 is shown above the alignment. Highly conserved residues predicted to be involved in the catalytic process are indicated in blue triangles. Black stars indicate the link between two parts of the DHH-DHHA1 domain.
(PPTX)

S4 Fig Enzymatic characterization of rMbovP328. Influence of temperature (A), pH values (B), divalent cations (C), and Mn^2+^ concentration (D) on the relative enzymatic activity of *M*. *bovis* rMbovP328. The phosphodiesterase activity of rMbovP328 was determined by HPLC analysis of c-di-AMP hydrolysis. Data shown in panels A to D are presented as the means values of three independent assays, with standard deviations indicated by error bars.
(PPTX)

S5 Fig PCR and RT-PCR analysis of *M*. *bovis* strains CNT9.386 and CNT9.386^H291A^. (A) PCR amplification of Mbov_0328 locus in *M*. *bovis* parental strain (HB0801), but not in mutant T9.386 (T9.386) having a mTn inserted in this region; and PCR amplification of the Mbov_0328 sequence encoded by plasmid pCN-T9.386 and pCN-T9.386^H291A^ in complemented strains CNT9.386 (CNT9.386) and CNT9.386^H291A^ (CNT9.386^H291A^), respectively. (B) RT-PCR amplification of Mbov_0328 transcripts in HB0801 (HB0801), complemented strains CNT9.386 (CNT9.386) and CNT9.386^H291A^ (CNT9.386^H291A^), but not in mutant T9.386 (T9.386). (C) Total RNA extracts from samples used for RT-PCR amplifications. DNA ladder (M) and negative control (-) are indicated.
(PPTX)

S6 Fig Venn diagrams of proteins identified in HB0801 and T9.386. Overlapping circles illustrating the number of proteins found repeatedly detected by LC-MS/MS in *M*. *bovis* populations grown in axenic conditions. (A) Analysis of proteins detected in triplicate samples of HB0801. (B) Analysis of proteins detected in triplicate samples of mutant T9.386. (C) Number of proteins found commonly expressed by HB0801 and T9.386.
(PPTX)

S1 Table Proteins differentially expressed in *M*. *bovis* mutant T9.386. (XLSX)

S2 Table DNA constructions, oligonucleotides, and recombinant proteins. (XLSX)

S1 Data Excel spreadsheet containing, in separate sheets, numerical values used to generate Figures. (XLSX)
