# Supplementary figures and images for: An emerging role for cyclic dinucleotide phosphodiesterase and nanoRNase activities in Mycoplasma bovis: Securing survival in cell culture
Source: PLoS Pathog. 2020 Jun 29;16(6):e1008661. doi: 10.1371/journal.ppat.1008661 (PMC7373297; doi:10.1371/journal.ppat.1008661)

## Slide 1
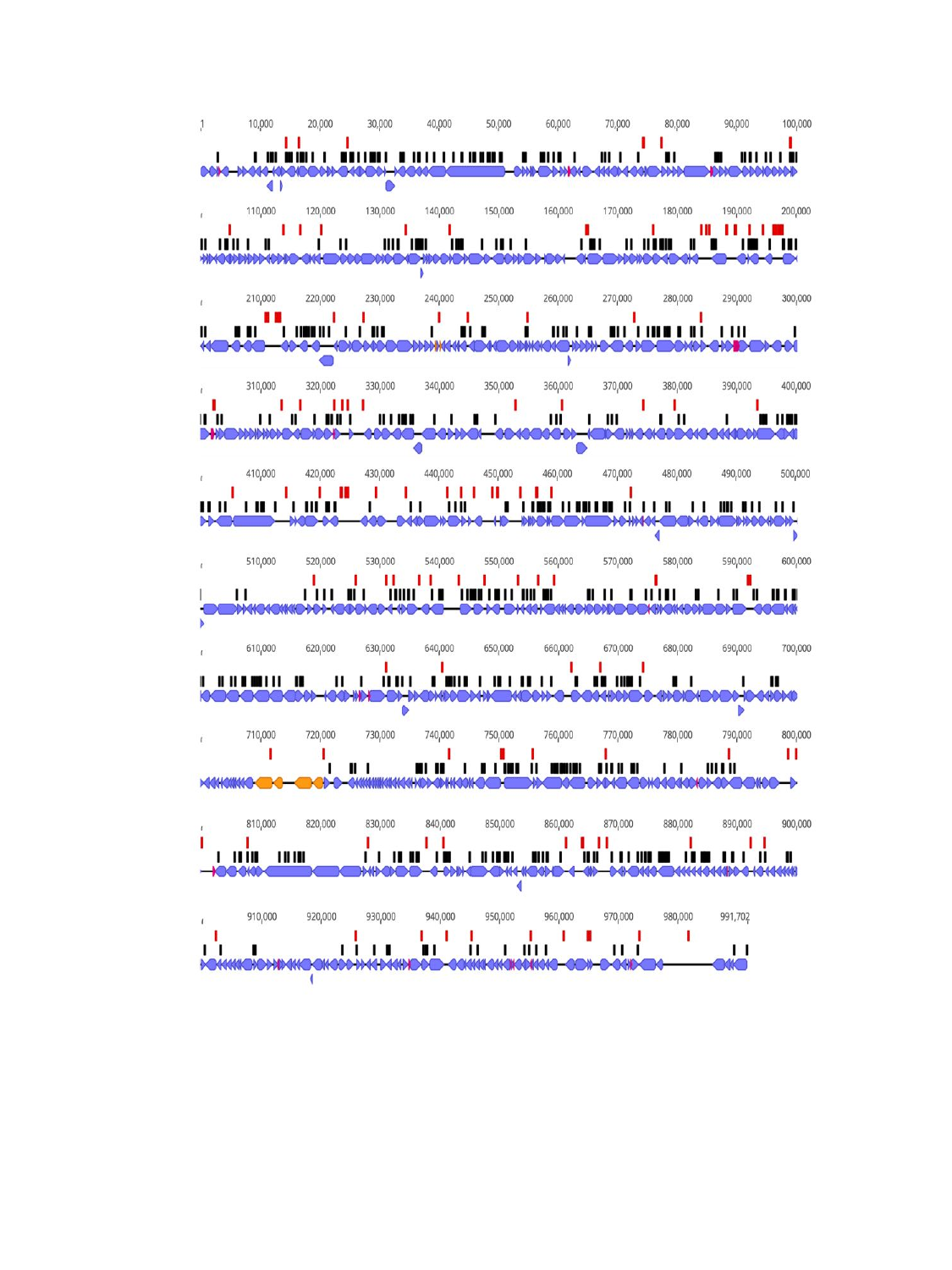

Supplement: S1 Fig — Schematic representation illustrating the distribution of unique mTn insertions in the HB0801 chromosome. Data are derived from sequence analysis of 1032 mutants. Transposons inserted into the coding sequences and non-coding regions are indicated by black and red lines, respectively. Coding sequences on the HB0801 chromosome are represented by blue arrows. Pink and orange colors indicate tRNA and rRNA regions, respectively. (PPTX) [file ppat.1008661.s001.pptx]

## Slide 1
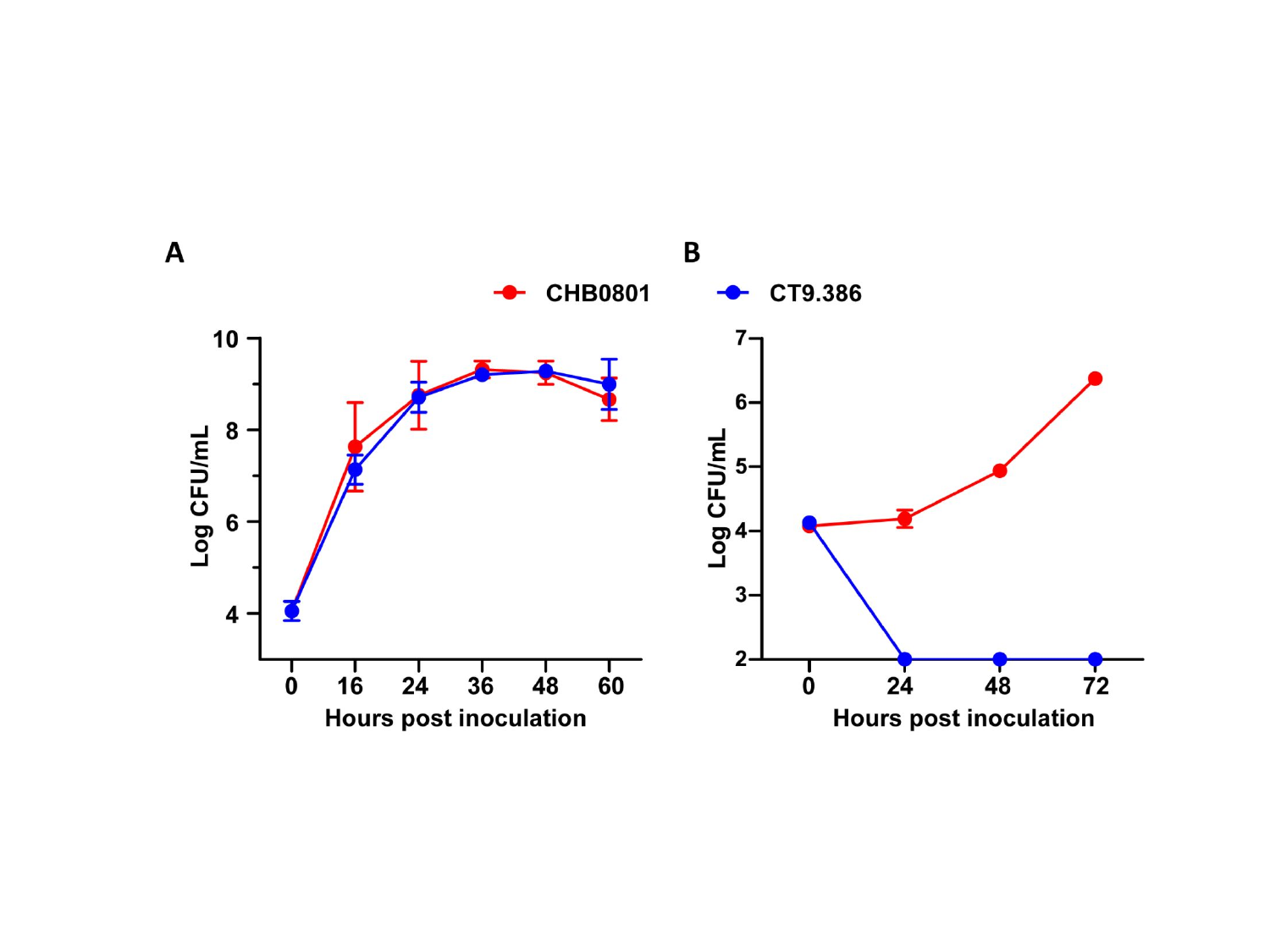

Supplement: S2 Fig — M. bovis CHB0801 and CT9.386 were generated following transformation of parental strain HB0801 and mutant T9.386 with the control plasmid pOH/P. (A) Growth phenotype of CHB0801 and CT9.386 in PPLO medium. Mycoplasmas (104 CFUs) were grown in 1 ml of PPLO medium. (B) Growth phenotype of CHB0801 and CT9.386 in cell culture. Mycoplasmas (104 CFUs/ml) were inoculated to EBL cells seeded at a density of 2 x 10^4^ cells/cm2. Mycoplasma titers were determined at different time post-inoculation. The data are presented as the means of three independent assays. Standard deviations are indicated by error bars. (PPTX) [file ppat.1008661.s002.pptx]

## Slide 1
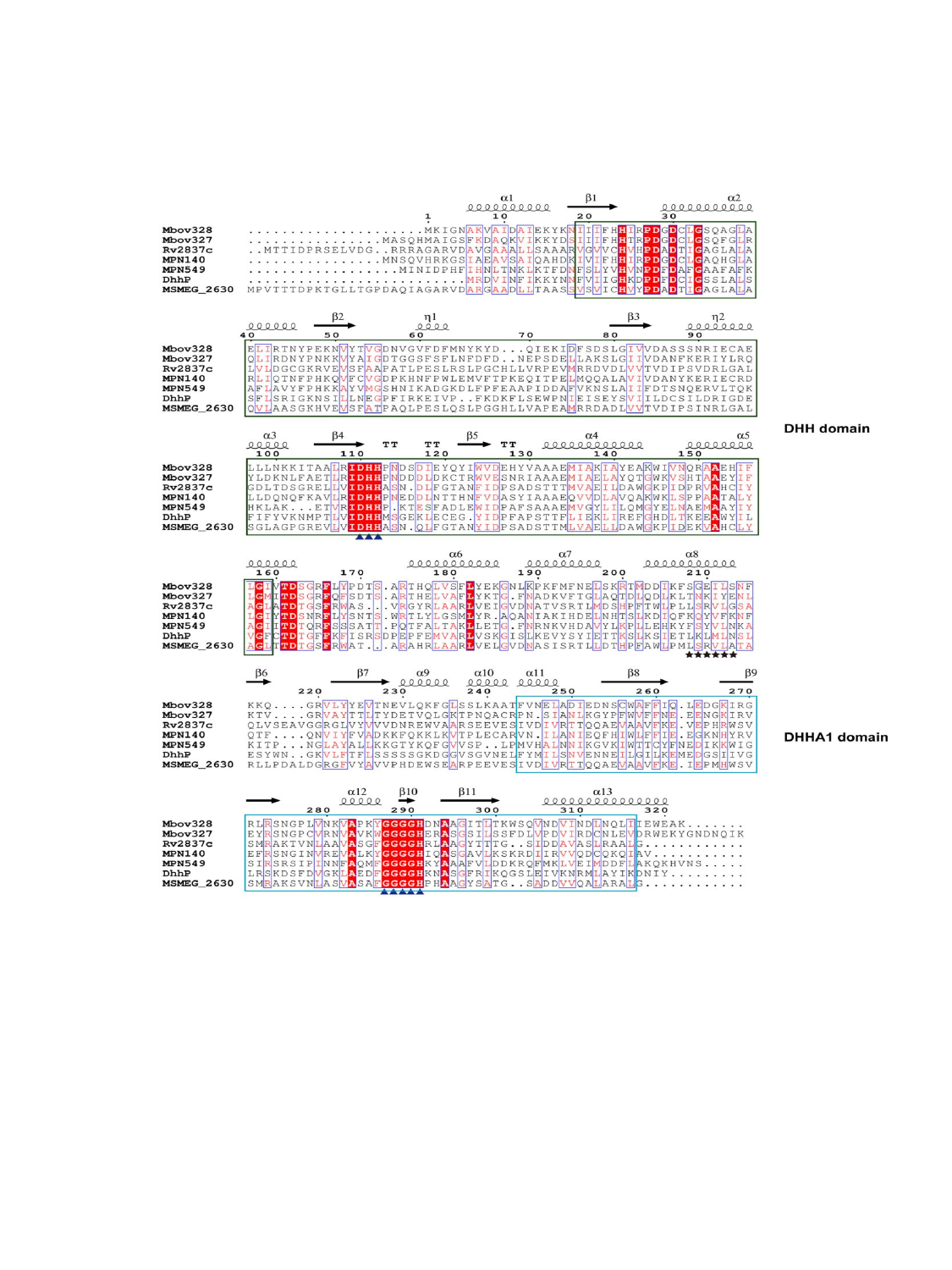

Supplement: S3 Fig — The alignments of M. bovis Mbov327 and Mbov328, Mycobacterium tuberculosis Rv2837c (PDB code 5CET), M. pneumoniae MPN140 (UniProtKB entry P75144) and MPN549 (UniProtKB entry P75229), Borrelia burgdorferi DhhP (UniProtKB entry O51564), and Mycobacterium smegmatis MSMEG_2630 (PDB code 4LS9) were performed using ESPript 3.0. The secondary structure of Mbov328 is shown above the alignment. Highly conserved residues predicted to be involved in the catalytic process are indicated in blue triangles. Black stars indicate the link between two parts of the DHH-DHHA1 domain. (PPTX) [file ppat.1008661.s003.pptx]

## Slide 1
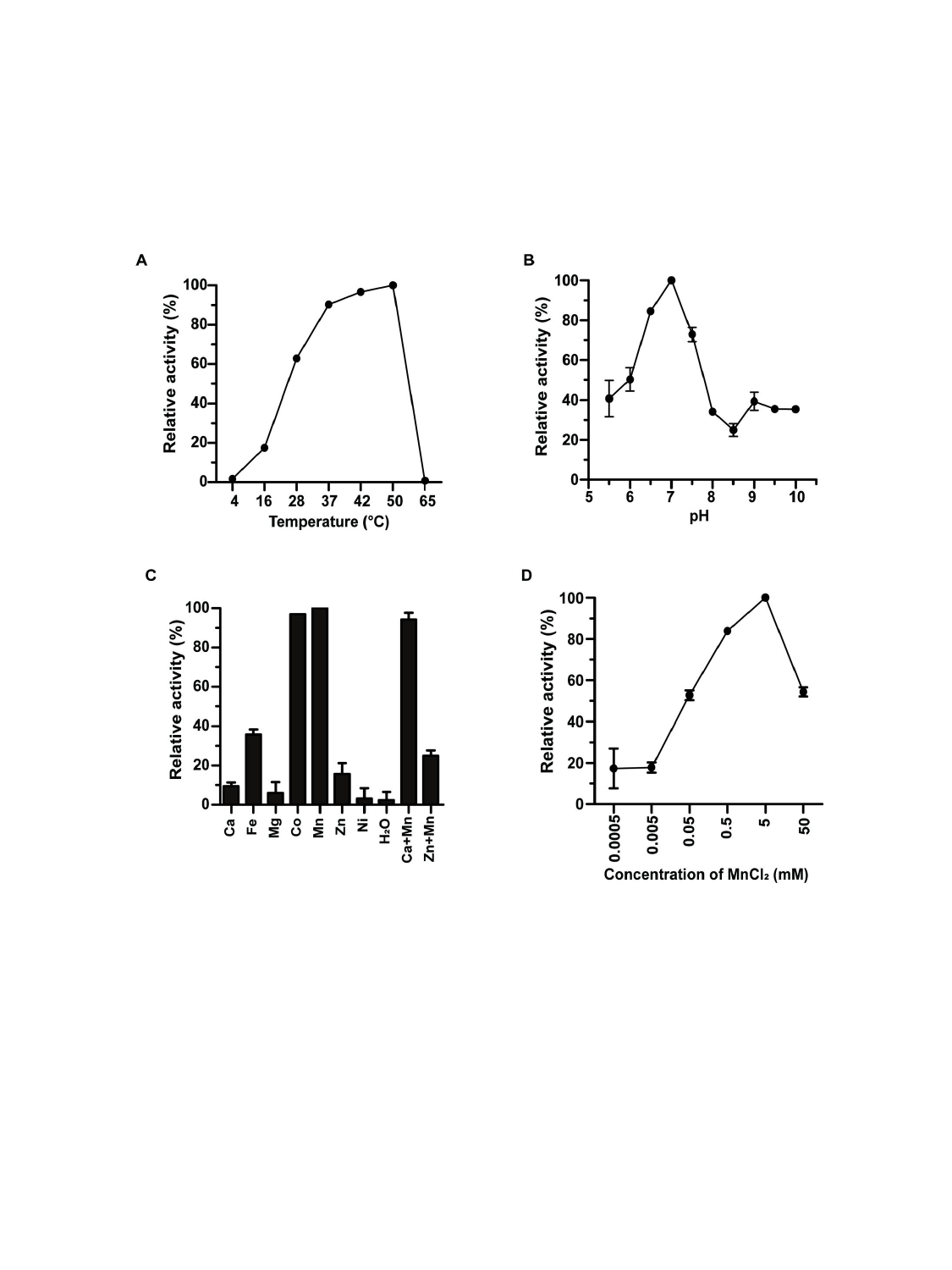

Supplement: S4 Fig — Influence of temperature (A), pH values (B), divalent cations (C), and Mn2+ concentration (D) on the relative enzymatic activity of M. bovis rMbovP328. The phosphodiesterase activity of rMbovP328 was determined by HPLC analysis of c-di-AMP hydrolysis. Data shown in panels A to D are presented as the means values of three independent assays, with standard deviations indicated by error bars. (PPTX) [file ppat.1008661.s004.pptx]

## Slide 1
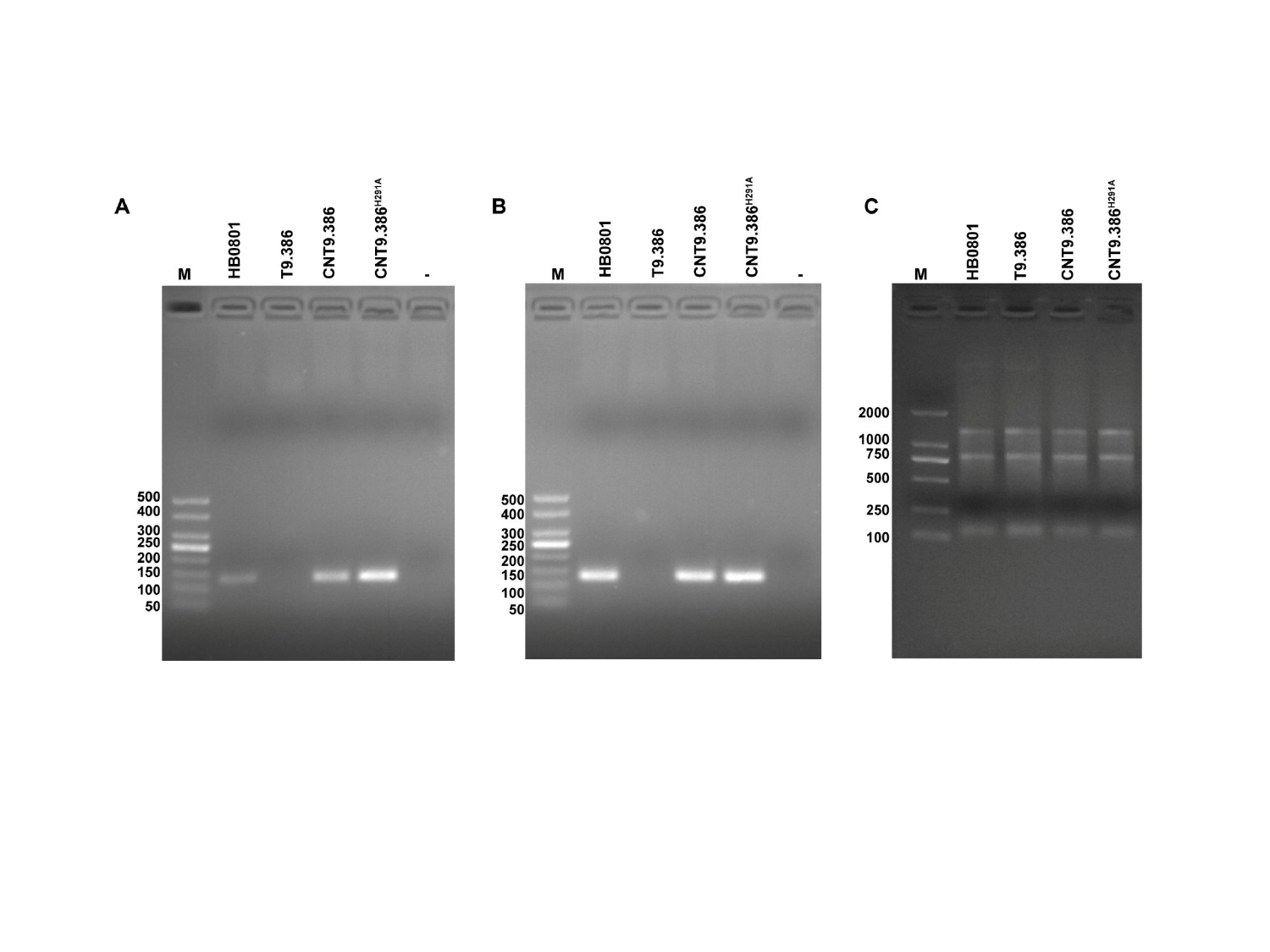

Supplement: S5 Fig — (A) PCR amplification of Mbov_0328 locus in M. bovis parental strain (HB0801), but not in mutant T9.386 (T9.386) having a mTn inserted in this region; and PCR amplification of the Mbov_0328 sequence encoded by plasmid pCN-T9.386 and pCN-T9.386H291A in complemented strains CNT9.386 (CNT9.386) and CNT9.386H291A (CNT9.386H291A), respectively. (B) RT-PCR amplification of Mbov_0328 transcripts in HB0801 (HB0801), complemented strains CNT9.386 (CNT9.386) and CNT9.386H291A (CNT9.386H291A), but not in mutant T9.386 (T9.386). (C) Total RNA extracts from samples used for RT-PCR amplifications. DNA ladder (M) and negative control (-) are indicated. (PPTX) [file ppat.1008661.s005.pptx]

## Slide 1
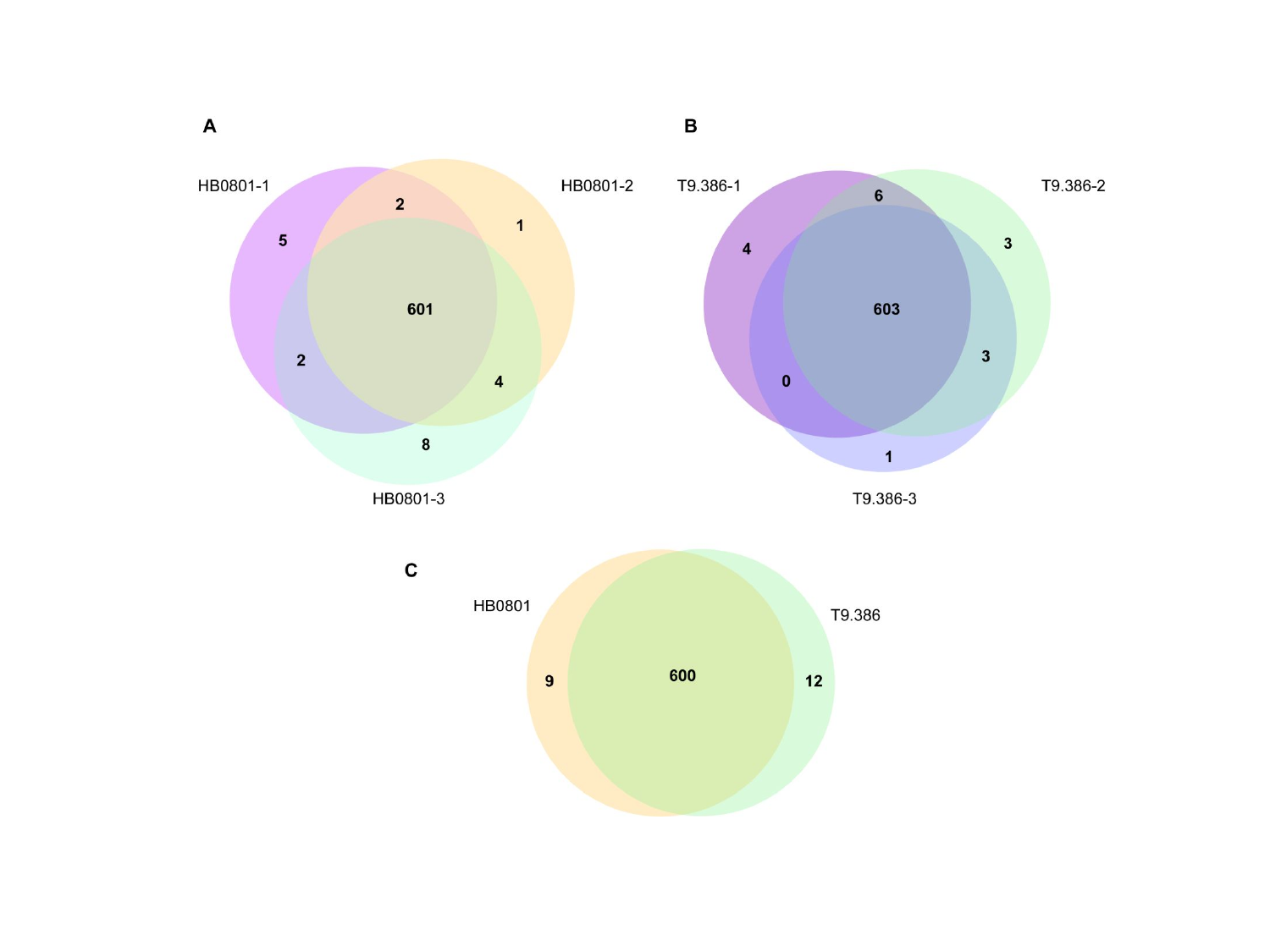

Supplement: S6 Fig — Overlapping circles illustrating the number of proteins found repeatedly detected by LC-MS/MS in M. bovis populations grown in axenic conditions. (A) Analysis of proteins detected in triplicate samples of HB0801. (B) Analysis of proteins detected in triplicate samples of mutant T9.386. (C) Number of proteins found commonly expressed by HB0801 and T9.386. (PPTX) [file ppat.1008661.s006.pptx]
